# Therapeutic alliance—not therapist competence or group cohesion—contributes to reduction of psychological distress in group‐based mindfulness‐based cognitive therapy for cancer patients

**DOI:** 10.1002/cpp.2352

**Published:** 2019-02-22

**Authors:** Else M. Bisseling, Melanie P.J. Schellekens, Philip Spinhoven, Félix R. Compen, Anne E.M. Speckens, Marije L. van der Lee

**Affiliations:** ^1^ Department of Psychiatry Radboud University Nijmegen Medical Centre for Mindfulness Nijmegen The Netherlands; ^2^ Donders Institute for Brain, Cognition and Behavior Radboud University Nijmegen The Netherlands; ^3^ Centre for Psycho‐Oncology, Scientific Research Department Helen Dowling Institute Bilthoven The Netherlands; ^4^ Institute of Psychology Leiden University Leiden The Netherlands; ^5^ Department of Psychiatry Leiden University Medical Center Leiden The Netherlands

**Keywords:** cancer, group cohesion, MBCT, psychological distress, therapeutic alliance, therapist competence

## Abstract

Mindfulness‐based cognitive therapy (MBCT) is an innovative evidence‐based intervention in mental and somatic health care. Gaining knowledge of therapeutic factors associated with treatment outcome can improve MBCT. This study focused on predictors of treatment outcome of MBCT for cancer patients and examined whether group cohesion, therapeutic alliance, and therapist competence predicted reduction of psychological distress after MBCT for cancer patients. Moreover, it was examined whether therapist competence facilitated therapeutic alliance or group cohesion. Multilevel analyses were conducted on a subsample of patients collected in a larger randomized controlled trial on individual internet‐based versus group‐based MBCT versus treatment as usual in distressed cancer patients. The current analyses included the 84 patients who completed group‐based MBCT out of 120 patients who were randomized to group‐based MBCT. Group cohesion and therapist competence did not predict reduction in psychological distress, whereas therapeutic alliance did. In addition, therapist competence did not predict therapeutic alliance but was associated with reduced group cohesion. Our findings revealed that therapeutic alliance significantly contributed to reduction of psychological distress in MBCT for cancer patients. Elaborating the clinical implications of the predictive significance of therapeutic alliance might be of added value to enhance the potential effect of MBCT.

**Key Practitioner Message**

- Mindfulness‐based cognitive therapy (MBCT) is an innovative evidence‐based intervention in mental and somatic health care and has been increasingly applied in oncology to reduce psychological distress.
- Therapeutic alliance predicts reduction in psychological distress after MBCT for cancer patients, whereas group cohesion and therapist competence did not.
- Therapist competence did not appear to be a precondition for a good therapeutic alliance and high group cohesion.
- Contrary to expectation, we found competence to be negatively related to group cohesion.
- Elaborating the clinical implications of the predictive significance of therapeutic alliance might be of added value to enhance the potential effect of MBCT for cancer patients.
- The current findings should be taken into account in the training of MBCT therapists.

## 1 BACKGROUND

Mindfulness‐based interventions (MBIs) are innovative evidence‐based interventions in mental and somatic health care (Gotink et al., 2015; Kuyken et al., 2016). MBIs have increasingly been applied in oncology. A 2012 meta‐analysis of nine randomized controlled trials (RCTs; *n =* 955) in cancer patients demonstrated that MBIs result in significant improvements in depressive and anxiety symptoms (Piet, Wurtzen, & Zachariae, 2012). Since then, several RCTs have confirmed these effects (Carlson et al., 2013; Compen et al., 2018; Garland et al., 2014; Johannsen et al., 2016; Lengacher et al., 2016). Mindfulness is defined as “paying attention; on purpose, in the present moment and non‐judgmentally” (Kabat‐Zinn, 2013). MBIs teach patients to become more aware of their experiences in daily life through meditation exercises, yoga, group discussion, and didactic teaching. MBI protocols designed to teach the cultivation of mindfulness are mindfulness‐based stress reduction (MBSR) and mindfulness‐based cognitive therapy (MBCT), the latter incorperating elements of cognitive behavioural therapy (CBT).

Research on predictors of treatment outcome focuses on both intrapsychological characteristics of participants and interpsychological therapeutic aspects, such as group cohesion, therapeutic alliance, and therapeutic competence. Independent of treatment orientation studies on therapeutic aspects accentuate the importance of these general factors (Ahn & Wampold, 2001; Messer & Wampold, 2002). Other studies indicate specific therapeutic factors to predict outcome of particular psychotherapies, such as embodiment as a key element of therapist competence in MBIs (van Aalderen, Breukers, Reuzel, & Speckens, 2014). Identifying therapeutic factors related to outcome may deepen our understanding of the processes that account for therapeutic change. Such knowledge can help to develop and test more precise treatment strategies that trigger critical change processes (Kazdin, 2008).

Group cohesion primarily refers to social–emotional cohesion: the intimacy, reciprocity, and emotional disclosure that is felt among group members (Yalom, 1995), contributing to the cooperation within the group to achieve a common therapeutic aim (Budman et al., 1989). It is presumed that when cohesion is high, the group is motivated to perform well and is more able to carry out activities for successful performance (Beal, Cohen, Burke, & McLendon, 2003). Research on the role of group cohesion in psychotherapy has shown that a higher level of group cohesion predicts better treatment outcome (Marziali, Munroe‐Blum, & McCleary, 1997; Taube‐Schiff, Suvak, Antony, Bieling, & McCabe, 2007; van Andel, Erdman, Karsdorp, Appels, & Trijsburg, 2003).

To measure group cohesion, the Dutch Group Cohesion Questionnaire‐22 (GCQ‐22; Trijsburg, Bogaerds, Letiche, Bidzjel, & Duivenvoorden, 2004) was developed, which is based on the Group Attitude Scale (Evans & Jarvis, 1986) and the Three Factor Group Questionnaire (Stokes, 1983). The GCQ‐22 compromises 22 items across four scales: (a) the bond with the group as total, (b) the bond with other members of the group, (c) the cooperation within the group, and (d) the instrumental value of the group.

To our knowledge, there are no previous studies on the impact of group cohesion in MBIs, whether in cancer patients or in different settings. However, qualitative studies showed that group‐based settings in MBIs are of added value to cancer patients (Mackenzie, Carlson, Munoz, & Speca, 2007; van Aalderen et al., 2014), and that peer support facilitated the learning process in MBSR (Schellekens et al., 2016).

Therapeutic alliance is defined as the collaborative and affective bond between therapist and patient (Bordin, 1994; Luborsky, 1994). Several meta‐analyses demonstrated the significant impact of therapeutic alliance on psychotherapy outcomes (Horvath, Del Re, Flückiger, & Symonds, 2011; Martin, Garske, & Davis, 2000). Therapeutic alliance is often measured with the Working Alliance Inventory (WAI; Horvath & Greenberg, 1989), including three subscales as follows: (a) how closely client and therapist agree on goals of treatment, (b) how closely client and therapist agree on how to reach the treatment goals, and (c) the degree of mutual trust between client and therapist. Only one small RCT examined the role of therapeutic alliance in MBCT (Snippe et al., 2015). Comparing individual CBT with individual MBCT, findings showed that therapeutic alliance predicted outcomes of CBT but not of MBCT in depressed patients with diabetes (Snippe et al., 2015).

Therapist competence refers to the level of therapist skills in delivering the treatment. It includes the therapists' consideration of and response to relevant contextual variables (Fairburn & Cooper, 2011; Waltz, Addis, Koerner, & Jacobson, 1993). Therapist competence has been shown to be associated with positive symptom change in CBT for patients with depressive and anxiety disorders (Ginzburg et al., 2012; Kuyken & Tsivrikos, 2009; Strunk, Brotman, DeRubeis, & Hollon, 2010; Webb, Derubeis, & Barber, 2010). However, recently, a large study (*n* = 1247) in routine clinical practice could not confirm this association (Branson, Shafran, & Myles, 2015).

The therapists' role is slightly different in MBCT compared with that of traditional CBT. In MBCT, for instance, therapist embodiment is considered to largely determine the quality of the therapy. Moreover, in MBCT, the therapist is mainly focused on facilitating patients' self‐efficacy, without too much emphasis on exploring personal narratives. To evaluate therapist competence, the Mindfulness‐Based Interventions–Teachers Assessment Criteria (MBI:TAC; Crane et al., 2012) is commonly used. The MBI:TAC consists of six domains. These domains consist of three to five key qualities that are scored by independent raters. Although these domains are considered important (Crane et al., 2012), only one multicentre study (*n* = 241) conducted on MBCT for recurrent depression elaborated upon teacher competence. In this study, no robust effects of teacher competence were found (Huijbers et al., 2017).

In conclusion, group cohesion and therapeutic alliance are closely related constructs, contributing independently to treatment outcome (van Andel et al., 2003). Therapeutic alliance and therapist competence are closely related as well. In some studies, it was found that therapeutic alliance mediates the relationship between competence and outcome in psychotherapy (Despland et al., 2009; Sharpless & Barber, 2009; Weck, Richtberg, Jakob, Neng, & Hofling, 2015) and was suggested that therapeutic competence can be seen as a precondition for developing a good therapeutic alliance. Put differently, only when the therapist is able to deliver the treatment competently, a good therapeutic alliance can be formed. However, conclusive evidence whether group cohesion, therapeutic alliance, and therapist competence predict treatment outcome is lacking.

The aim of our explorative study was to examine the role of group cohesion, therapeutic alliance, and therapist competence on outcome of MBCT for distressed cancer patients. It was hypothesized that (a) group cohesion, therapeutic alliance, and therapist competence independently predict reduction of psychological distress, and (b) therapist competence is a precondition for developing both group cohesion and therapeutic alliance which, in turn, independently predict reduction of psychological distress.

## 2 METHODS

### 2.1 Design

The present study was part of a larger multicentre RCT on the effectiveness of MBCT for distressed cancer patients (Clinicaltrials.gov no. NCT02138513; Compen et al., 2015). Participants were randomized to either (a) face‐to‐face group MBCT, (b) online individual MBCT (eMBCT), or (c) treatment as usual (TAU). After 3 months, patients in TAU were randomly allocated to (a) face‐to‐face group‐based MBCT or (b) eMBCT. For the present study, we used the data of 120 patients who were allocated to the face‐to‐face group‐based MBCT after the initial randomization (*n* = 77) or after the completing the TAU condition (*n* = 43). The analyses only included patients who completed the group‐based MBCT, that is, attended four or more sessions (*n* = 84). The local ethics committee approved this study (CMO Arnhem Nijmegen 2013/542). All patients and therapists provided written informed consent.

### 2.2 Study population

#### 2.2.1 Patients

Patients were recruited in participating specialized mental health care institutes for psycho‐oncology, via social media, patient associations, and advertorials in local newspapers in the Netherlands. Patients who were interested in participation could enrol themselves at the study website (www.bemind.info) at which point they completed the screening assessment, the Hospital Anxiety and Depression Scale (HADS). Patients with a score of ≥11 on the HADS were contacted by telephone by one of the researchers to assess eligibility. Inclusion criteria were having any cancer diagnosis, experiencing at least mild psychological distress, sufficient computer literacy and access to internet, good command of Dutch language, and willingness to participate in either online or face‐to‐face group‐based MBCT. Exclusion criteria were severe psychiatric morbidity such as suicidal ideation and/or psychosis, change in psychotropic medication within 3 months of baseline, and current or previous participation in MBCT or MBSR. Prior to randomization, patients completed the baseline assessment and (self‐report) questionnaires, including the following demographic and clinical characteristics: gender, age, marital status, children, education, cancer diagnosis, and years since diagnosis, anticancer treatment intent, current anticancer treatment, and psychiatric diagnosis.

#### 2.2.2 Therapists

Therapists (*n* = 9) were affiliated to the participating centres, including specialized outpatients clinics for psycho‐oncology (*n* = 4), a general and an academic hospital (*n* = 3), and private practices (*n* = 2). All therapists fulfilled the advanced criteria of the Association of Mindfulness‐Based Teachers in the Netherlands and Flanders that are in concordance with the UK Mindfulness‐Based Teacher Trainer Network Good Practice Guidelines for teaching mindfulness‐based courses (Crane et al., 2012). These include a minimum of 150‐hr education in MBSR/MBCT, at least 3 years of personal practice of meditation, having attended at least one 5–10 days retreat, and teaching a minimum of two MBCT trainings a year. The supervision offered within the multicentre trial was on a national level. All therapists received a two‐day workshop and additional training in the MBCT study protocol by experienced senior psychologists and psychiatrists who were also mindfulness teachers. Two additional supervision meetings were organized during the intervention phase that lasted 1 year and 6 months. In addition, all therapists received regular supervision at their individual working places. When facing difficulties, therapists were encouraged to contact one of the researchers, who were experienced psychiatrists and mindfulness trainers.

### 2.3 Intervention

#### 2.3.1 Mindfulness‐based cognitive therapy

Patients randomized to group‐based MBCT received the intervention according to the MBCT protocol of Segal, Williams, and Teasdale (2013). The group‐based MBCT consisted of eight weekly 2.5‐hr group sessions, one 6‐hr silent day, and daily home practice assignments guided by audio files. The sessions consisted of mindfulness practices, sharing experiences, and didactic teachings, which were adapted to cancer patients (Compen et al., 2015). Each participant received a folder with information on each session and a compact disc containing the audio files. The group‐based MBCT was provided at the Radboud University Medical Centre in Nijmegen, the Jeroen Bosch Hospital in 's‐Hertogenbosch and at four mental health institutes specialized in psycho‐oncology (Helen Dowling Institute [Bilthoven], Ingeborg Douwes Centrum [Amsterdam], De Vruchtenburg [Leiden], and Het Behouden Huys [Haren]). In the group‐based MBCT condition, all sessions of all therapists were videotaped to evaluate teacher competence and protocol adherence. In total, 14 face‐to‐face MBCT groups were delivered. The intervention is described in more depth in our protocol paper (Compen et al., 2015).

### 2.4 Measures

#### 2.4.1 Treatment outcome

The primary outcome measure was psychological distress according to the 14‐item HADS (Spinhoven et al., 1997; Zigmond & Snaith, 1983). It has been validated in somatic patient populations, including cancer patients (Bjelland, Dahl, Haug, & Neckelmann, 2002). Internal consistency of the total scale in the present sample was high (Cronbach's *α* = 0.82).

#### 2.4.2 Therapeutic factors

Group cohesion was assessed with the Dutch GCQ‐22 (Trijsburg et al., 2004), which has been used in cancer patients before (May et al., 2008). The Dutch GCQ consists of four subscales as follows: (a) the bond with the group as whole, (b) the bond with other members, (c) cooperation within the group, and (d) the instrumental value of the group bond. Each item of this 22‐item inventory is rated from 1 (*totally disagree*) to 6 (*totally agree*). Internal consistency of total scale of the version used in this study was 0.95. In this study, the GCQ was administered at the start of Session 5, in accordance with a previous study in cancer patients, suggesting group cohesion to develop early in the intervention and to stay relatively stable over time (May et al., 2008). Due to the layout of the GCQ Teleform, the percentage of missing values on the last three items was 24%. These missing values were replaced with mean values of the available items.

Therapeutic alliance was measured with a translated and shortened form of the WAI (Horvath & Greenberg, 1989), which was administered at the start of Session 5. The WAI consists of three subscales assessing: (a) how closely client and therapist agree on and are mutually engaged in the goals of treatment; (b) how closely client and therapist agree on how to reach the treatment goals; and (c) the degree of mutual trust, acceptance, and confidence between client and therapist. Items were scored on a 5‐point scale ranging from *rarely* to *always* (Hatcher & Gillaspy, 2006; Stinckens Ulburghs, & Claes, 2009). The 12‐item inventory was validated in a Dutch‐speaking sample, showing an internal consistency of >0.80 for all separate subscales and 0.87 for the total scale (Janse, Boezen‐Hilberdink, van Dijk, Verbraak, & Hutschemaekers, et al., 2014). Internal consistency of the total scale of the version used in this study was 0.87.

Therapist competence was assessed with the MBI:TAC (Crane et al., 2012) that consists of six domains: (a) organization, (b) relational skills, (c) embodiment of mindfulness, (d) guiding mindfulness practices, (e) didactical skills, and (f) group environment. These domains consist of three to five key qualities that are scored at six competence levels: (a) incompetent, (b) beginner, (c) advanced beginner, (d) competent, (e) proficient, and (f) advanced. Therapist competency levels were determined for all nine therapists providing face‐to‐face MBCT. From each therapist, two videotaped sessions were randomly selected (www.random.org). The sessions were rated by two independent raters who were both experienced mindfulness teachers. Both assessors had not participated in the RCT as a therapist and had experience with assessing competence using the MBI:TAC. To assess the interrater reliability of the MBI:TAC, intraclass correlations were calculated using a two‐way mixed consistency model with single measures, on the basis of the independent ratings of two assessors per videotape (*n* = 5). The IRR was substantial with an ICC score of the total scale of 0.70. The ICC's of the six domains were as follows: fair for guiding practices (0.27), moderate for organization (0.59), substantial for relational skills (0.78), embodiment (0.71), group environment (0.79), and almost perfect for didactical skills (0.84). Correlations between the domains were high, ranging from 0.72 to 0.96 (all *p* values < 0.05).

### 2.5 Statistical analyses

All analyses were conducted in SPSS Version 25.0. (IBM Corp, 2017) using an intervention completer sample. To examine possible predictors (group cohesion, therapeutic alliance, and therapist competence) of the effect of MBCT on psychological distress, multilevel analyses were used, in which participants were nested within therapist. We ran separate models for each predictor. The posttreatment HADS score was the dependent variable, and baseline HADS score was added as a covariate and the predictor of choice as a fixed factor. In addition, intercepts were allowed to vary across therapists. An unstructured covariance structure was used with no constraints or patterns specified in the covariance matrix. As the sample size was relatively small, restricted maximum likelihood was used to handle missing data (Newman, 2014). In addition, we explored whether therapist competence was a predictor for developing both group cohesion and therapeutic alliance. We used similar multilevel models adding competence as the predictor and group cohesion or therapeutic alliance as dependent variable. When the therapeutic factor was a significant predictor of outcome, we performed exploratory analysis to examine the effects of the subscales of that particular factor on outcome. Similar multilevel models were used, adding all subscale scores of the significant factor in one model.

Patients filled out the HADS prior to randomization (baseline, T0), directly after the intervention (T1), or directly after TAU (T0b). After TAU, the scores on the HADS in the TAU condition had not significantly changed (Compen et al., 2018). For patients participating in the group‐based MBCT after TAU, baseline scores on the HADS were replaced with end‐of‐waiting list scores as those were closer in time to the start of the treatment, in accordance with a previous study on the long‐term effect of (e)MBCT in cancer patient (Cillessen et al., 2018). These scores did not significantly differ from baseline scores of the original intervention group. Only three out of *n* = 25 (12%) of the completers, who started the intervention after TAU, scored <11 on the HADS at the start of the intervention. Analyses were also performed with only individuals scoring above the cut‐off of ≥11 on the HADS. These analyses showed similar results.

## 3 RESULTS

### 3.1 Participants

#### 3.1.1 Patients

**Figure 1:**
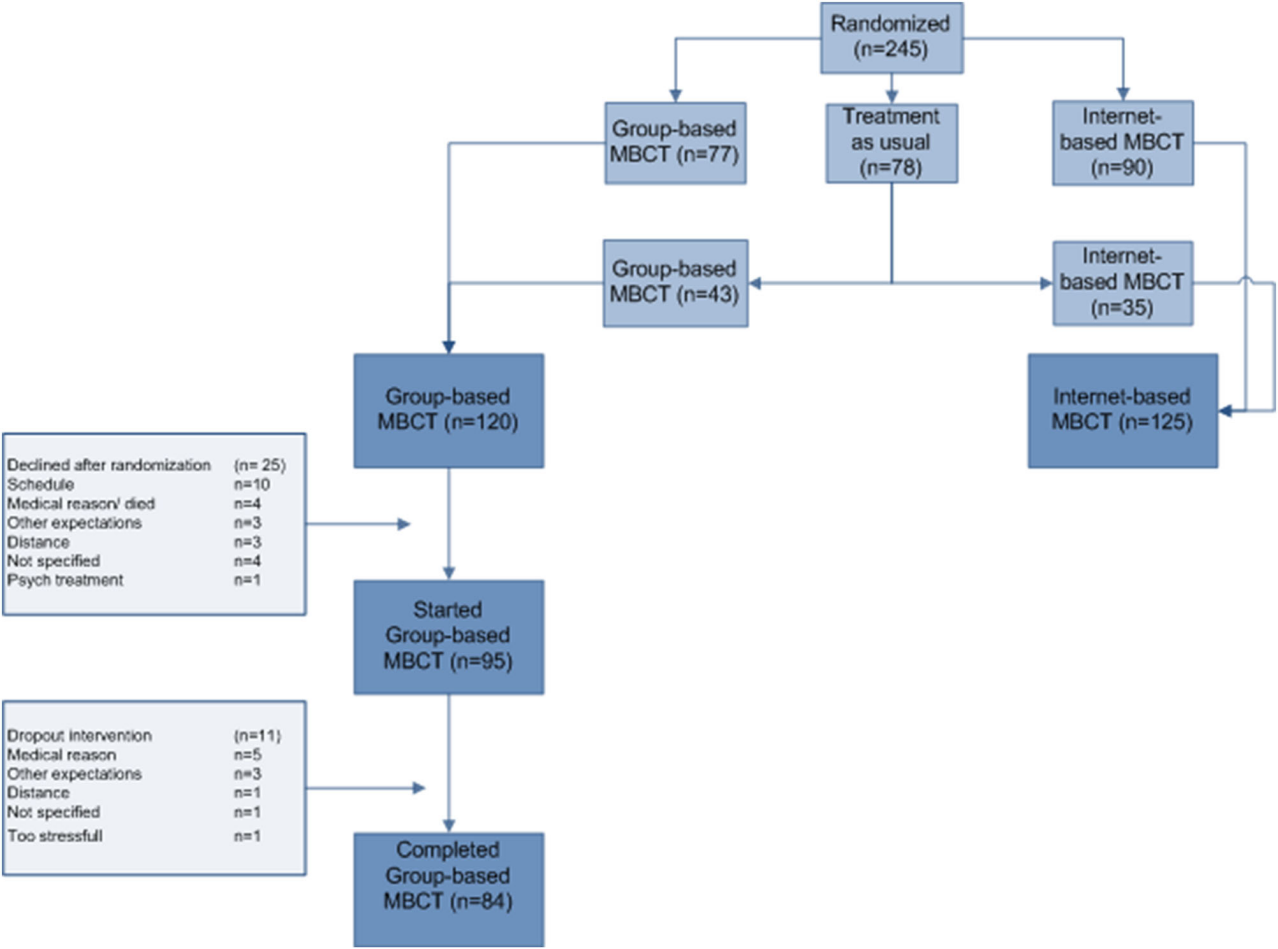
Study‐flow describing the composition of the subsample of *n* = 120 patients who received group‐based mindfulness‐based cognitive therapy (MBCT) [Colour figure can be viewed at wileyonlinelibrary.com]

Of the cancer patients participating in the RCT (*n* = 245), 120 were randomized to the face‐to‐face group MBCT: *n =* 77 starting immediately and *n =* 43 after the TAU condition. Of those, *n =* 25 (21%) decided not to take part after all, and *n =* 95 (79%) actually started with MBCT (see Figure 1 for the study flow diagram). Of all individuals randomized to group‐based MBCT, *n =* 84 (70%) completed more than four sessions of MBCT. As shown in Table 1, patients were mostly female, middle aged, highly educated, suffered from breast cancer, and were treated with a curative intent. There were no significant differences between the patients who completed the MBCT and those who did not start or dropped out the intervention. Multilevel analyses revealed that psychological distress, *F* (1, 74) = 72.11, *p* < 0.001, was significantly less at post‐treatment compared with baseline.

**Table 1:** Demographical and clinical characteristics of MBCT patients (*n* = 120)

			Completer (*n* = 84)	Decliner/dropout (*n* = 36)	Test statistic (*t* or *χ* ^2^)	*p*
Sociodemographic	Age *M* (*SD*)	Years	52.6 (10.7)	49.2 (11.9)	1.5	0.127
	Gender *n* (%)	Female	70 (83.3)	31 (86.1)	1.5	0.702
	Educational level *n* (%)	Secondary	25 (29.8)	10 (27.3)	0.48	0.827
		Vocational/university	59 (70.2)	26 (72.7)		
Clinical	Cancer diagnosis *n* (%)	Breast	54 (64.3)	21 (58.3)	0.38	0.537
		Prostate	7 (8.3)	1 (2.8)		
		Colon	5 (6.0)	3 (8.3)		
		Gyn.	4 (4.8)	1 (2.8)		
		Non‐Hodgkin	2 (2.4)	3 (8.3)		
		Other	12 (14.3)	7 (19.4)		
	Time since diagnosis *M* (*SD*)	Years	3.7 (5.3)	3.0 (4.1)	0.67	0.506
	Cancer treatment intent *n* (%)	Curative	74 (88.1)	30 (83.3)	0.5	0.482
	Current anticancer treatment *n* (%)	Yes	41 (48.8)	15 (41.7)	0.52	0.472
Treatment outcome	Psychological distress *M* (*SD*)	HADS	18.3 (6.5)	18.0 (7.0)	0.22	0.826
Process factor	Therapeutic alliance *M* (*SD*)	WAI	39.1 (8.8)			
	Group cohesion *M* (*SD*)	GCQ	95.3 (11.3)			

*Note*. GCQ: Group Cohesion Questionnaire; HADS: Hospital Anxiety and Depression Scale; MBCT: mindfulness‐based cognitive therapy; WAI: Working Alliance Inventory.

#### 3.1.2 Therapists

All therapists were middle‐aged females, with a mean duration of teaching MBCT of 6.1 years (*SD* = 2.9; *Mdn* = 7) and an average number of 26 courses taught (*SD* = 28.6; *Mdn* = 12). Of the nine rated therapists, two therapists were rated as beginner (22*%*), three as competent (33*%*), and four as proficient (44*%*) on the basis of the mean scores of the subscales. No therapist was rated as incompetent. Table 2 shows further details of therapist competence.

**Table 2:** Demographical characteristics and competence levels of MBCT therapist (*n* = 9)

		*M*	*SD*	Range	*N*	%
Age	Years	52.9	5.3	42–60		
Gender	Female				9	100%
Experience teaching MBCT	Years	6.1	2.9	2–10		
Experience teaching MBCT	Lifetime number of groups	25.9	28.6	8–91		
Level of competence	MBI:TAC total	4.1	0.8	1.8–5		
	MBI:TAC organization	3.5	1.0	1–5		
	MBI:TAC relational skills	4.8	1.0	2–6		
	MBI:TAC embodiment	4.1	1.1	2–6		
	MBI:TAC guiding practices	3.9	0.9	2–5		
	MBI:TAC didactical skills	4.1	1.1	1–5		
	MBI:TAC group environment	4.3	0.9	2–5		

*Note*. MBCT: mindfulness‐based cognitive therapy; MBI:TAC: Mindfulness‐Based Interventions–Teachers Assessment Criteria.

#### 3.1.3 Prediction of treatment outcome

Mean level of psychological distress was reduced from 18.3 (*SD* = 6.5) to 13.5 (*SD* = 6.8) at post‐treatment. Mean level of group cohesion at the start of Session 5 was 95.3 (*SD* = 11.3) and mean level of therapeutic alliance 39.1 (*SD* = 8.8). As Table 3 demonstrates, group cohesion did not significantly predict reduction of psychological distress (*b* = −0.10, *p* = 0.058), whereas therapeutic alliance did (*b* = −0.18, *p* = 0.016). As the therapeutic alliance significantly predicted reduction of psychological distress, we further explored potential effects of the three subscales. Exploratory analyses suggested significant predictive values of the goal subscale (*b* = −0.53, *p* = 0.010) but not of the task subscale (*b* = −0.08, *p* = 0.552) or the bond subscale (*b* = −0.19, *p* = 0.382). Therapist competence did not predict reduction of psychological distress (*b* = −0.10, *p* = 0.883). Therapist competence and therapeutic alliance were not significantly correlated (*r* = −0.206, *p* = 0.103). In addition, therapist competence was not associated with level of therapeutic alliance (*b* = −2.16, *p* = 0.120). However, therapist competence was inversely correlated (*r* = −2.83, *p* = 0.018) with group cohesion. Therapist competence appeared to be negatively associated with lower group cohesion (*b* = −3.90, *p* = 0.018), as shown in Table 4. Moreover, analyses revealed that group cohesion and therapeutic alliance were moderately correlated with one another (*r* = 0.515, *p* < 0.001). Exploratory analyses of the six domains of therapist competence suggested that the negative association with group cohesion was mainly due to the significant negative association with the domain relational skills (*b* = −3.58, *p* = 0.012). The other domains were not significantly associated with group cohesion.

**Table 3:** Group cohesion (A), therapeutic alliance (B), and competence (C) as predictor of treatment outcome

	*B*	*SE*	95% CI	*t*	*p*
A. Group cohesion as predictor of treatment outcome of MBCT					
Intercept	10.68	5.19	[0.27, 21.1]	2.06	0.045
Baseline level of outcome	0.73	0.09	[0.55, 0.91]	7.93	<0.01
Group cohesion	−0.10	0.53	[−0.21, 0.01]	−1.94	0.058
Group subscale	−0.36	0.15	[−0.66, −.06]	−2.38	0.020*
Member subscale	−0.02	0.18	[−0.39, 0.35]	−0.10	0.918
Cooperation subscale	−0.41	0.25	[−0.92, 0.10]	−1.60	0.113
Instrumental subscale	−0.33	0.12	[−0.57, −0.09]	−2.75	0.008*
B. Therapeutic alliance as predictor of treatment outcome of MBCT					
Intercept	9.90	3.87	[2.08, 17.7]	2.55	0.014
Baseline level of outcome	0.63	0.09	[0.44, 0.82]	6.63	<0.001
Therapeutic alliance	−0.18	0.71	[−0.32, −0.03]	−2.50	0.016*
Goal subscale	−0.53	0.20	[−0.92, −0.13]	−2.68	0.010*
Task subscale	−0.08	0.12	[−0.33, 0.18]	−0.60	0.552
Bond subscale	−0.19	0.22	[−0.62, 0.24]	−0.88	0.382
C. Therapist competence as predictor of treatment outcome of MBCT					
Intercept	0.70	3.29	[−6.31, 7.72]	0.21	0.833
Baseline level of outcome	0.74	0.09	[0.57, 0.92]	8.45	<0.001
Therapist competence	−0.10	0.68	[−1.68, 1.48]	−0.15	0.833
Coverage	0.21	0.64	[−1.41, 1.84]	0.33	0.752
Relational skills	−0.16	0.61	[−1.59, 1.27]	−0.26	0.802
Embodiment	−0.16	0.55	[−1.57, 1.25]	−0.29	0.783
Guiding mindfulness	−0.01	0.70	[−1.67, 1.65]	−0.15	0.988
Didactic teaching	−0.01	0.55	[−1.29, 1.28]	−0.02	0.987
Group environment	−0.37	0.63	[−1.86, 1.11]	−0.58	0.576

*Note*. Total scale and subscale analyses were performed in separate multilevel analyses. MBCT: mindfulness‐based cognitive therapy.

* *p* = <0.05.

**Table 4:** Therapist competence as predictor of therapeutic alliance (A) and group cohesion (B)

	*B*	*SE*	95% CI	*t*	*p*
A. Therapist competence as predictor of therapeutic alliance					
Intercept	48.2	5.61	[36.4, 60.0]	8.59	<0.001
Therapist competence	−2.16	1.32	[−4.95, 0.63]	−1.64	0.120
Coverage	−2.33	1.36	[−5.34, 0.67]	−1.72	0.115
Relational skills	−2.1	1.12	[−4.35, 0.15]	−1.87	0.066
Embodiment	−1.39	1.07	[−3.87, 1.07]	−1.31	0.229
Guiding mindfulness	−1.63	1.35	[−4.66, 1.39]	−1.21	0.256
Didactic teaching	−1.55	1.13	[−3.95, 0.84]	−1.38	0.188
Group environment	−1.63	1.31	[−4.44, 1.19]	−1.24	0.235
B. Therapist competence as predictor of group cohesion					
Intercept	111	6.87	[97.9, 125]	16.25	<0.001
Therapist competence	−3.90	1.61	[−7.11, 0.68]	−2.42	0.018*
Coverage	−3.44	1.59	[−7.01, 0.12]	−2.163	0.056
Relational skills	−3.58	1.39	[−6.34, −0.81]	−2.58	0.012*
Embodiment	−2.67	1.37	[−5.81, 0.48]	−1.94	0.086
Guiding mindfulness	−3.18	1.71	[−7.05, 0.70]	−1.85	0.097
Didactic teaching	−2.87	1.37	[−5.84, 0.10]	−2.10	0.057
Group environment	−2.72	1.62	[−6.24, 0.80]	−1.68	0.118

*Note*. Total scale and subscale analyses were performed in separate multilevel analyses.

* *p* = <0.05.

## 4 DISCUSSION

The present study investigated the association of group cohesion, therapeutic alliance, and therapist competence with reduction of psychological distress after MBCT for cancer patients. It was hypothesized that group cohesion, therapeutic alliance, and therapist competence would predict treatment outcome, and that therapist competence was a prerequisite for developing both group cohesion and therapeutic alliance. Our findings revealed that therapeutic alliance and not group cohesion significantly contributed to reduction of psychological distress in MBCT for cancer patients. Therapist competence did not predict post‐treatment psychological distress and did not appear to be a precondition for a good therapeutic alliance and high group cohesion.

In accordance with comprehensive and consistent findings in meta‐analyses in psychotherapy (Horvath et al., 2011; Martin et al., 2000), therapeutic alliance was positively associated with treatment outcome in our study as well, particularly in terms of goal aspects. This is in line with previous research but contrary to findings of one recent study investigating the relationship between therapeutic alliance and treatment outcomes in individual MBCT. In this study, no significant association with dimensions of the alliance were found (Snippe et al., 2015). Snippe et al. suggested that in general the low predictive value of task and goals aspects in MBCT could be explained by the experiential nature of MBCT. In contrast with this study, our results suggest that although MBCT focuses on experiential learning, still mutual agreement on the goals of the treatment is associated with a positive outcome. This has previously been established in CBT (Salvio, Beutler, Wood, & Engle, 1992; Spinhoven, Giesen‐Bloo, van Dyck, Kooiman, & Arntz, 2007).

Moreover, we did not find a significant association of group cohesion with symptom reduction. This is in line with a previous study showing that only the cooperation subscale, yet not the total scale, of the GCQ was positively correlated with outcome in a rehabilitation programme for cancer patients (May et al., 2008). In a well‐structured setting, group cohesion might be of less importance to acquire certain skills.

Therapist competence did not have a significant effect on reduction of psychological distress. This is in line with previous findings that did not show a relationship between therapist competence and treatment outcome in MBCT for recurrent depression (Huijbers et al., 2017). In addition, previous research in CBT also found little support for an association between competence and patient outcome (Branson et al., 2015). An explanation could be that all therapists operated at least at a basic skill level as they all fulfilled the advanced criteria of the Association of Mindfulness‐Based Teachers in the Netherlands and Flanders. Although there was a range in competence level, none of the therapists were observed as incompetent or advanced on the MBI:TAC. Therefore, differences in competence might have been too small to find an association with outcome. Therapist competence did not predict therapeutic alliance either. This could be explained by the finding that valued aspects of observed competence, such as a nonreacting stance, are not necessarily seen as positive by patients (van Aalderen et al., 2014). Personal style differences of therapists could have had a negative or positive impact on perceived therapeutic alliance from patients' perspective, irrespective of their competence. A second explanation could be that the highly structured programme, the pre‐recorded audio files, and the emphasized self‐efficacy may be predominant. These aspects should be further explored. From a more methodological view, therapist competence was assessed by observers, and both therapeutic alliance, group cohesion, and psychological distress were self‐reported by patients. In other words, the association between therapeutic alliance and group cohesion and outcome was based on data of a common source (e.g., therapeutic alliance from patients predicts psychological distress from patients), whereas the association between therapist competence and outcome was based on data of different sources (e.g., competence from therapists predicts psychological distress from patients). Due to shared method variance (Orth, 2013), chances of finding an effect based on data of a common source (patients effects) are higher than finding an effect based on different sources (therapist effect), and this might have influenced our results.

Contrary to our expectations, therapist competence appeared to be associated with lower group cohesion, mainly due to the significant negative association with the domain “relational skills.” In the observer‐rated MBI:TAC, relational skills refer to skills in managing a group learning environment, such as managing issues as ground rules, boundaries and confidentiality, and to leadership style. In contrast, the self‐rated GCQ mainly focuses on social–emotional aspects that are felt among group members within the group as a whole. It seems that the more skilled the MBCT therapist is in managing a group learning environment, the less patients need to rely on their fellow participants for support. Another explanation could be that competent therapists might focus more on guiding the exercises and conducting the inquiry, whereas less competent therapists might leave more room for the casual exchange of individual experiences enhancing group cohesion. An alternative explanation could be that independent raters observing video fragments have a different perspective on relational skills of the MBCT teacher than the MBCT participants themselves. More research is needed to examine these relational aspects.

This is the first study that examined the concurrent effect of group cohesion, therapeutic alliance, and therapist competence on outcome of MBCT. The strength of this study was that we rigorously observed therapist competence by two independent experienced raters with a measure developed to assess competence of mindfulness therapists. A few limitations need to be mentioned as well. First, following from the aim of the study, its design was uncontrolled. We could not perform a formal mediation analysis because we could not measure therapist competence or therapeutic alliance in a control group, nor could we measure group cohesion in the individual internet‐based MBCT. As there is no competence measure for internet‐based MBCT, we could not measure therapist competence in this intervention either. Furthermore, our sample size was small, and the diversity in competence of the therapists was limited. Different results might be found in a larger group of therapists with a broader range of competence, although this might be difficult to achieve in clinical trials. Moreover, patients could enrol themselves in the study. This patient‐centred nature of recruitment resulting in a convenience sample might benefit generalization of research findings to clinical practice but inherently leads to selection bias.

Further, research examining the effect of competence on outcome in MBCT should consider the use of data from different perspectives, such as therapist competence as perceived by patients. In addition, it should elaborate clinical implications of the predictive significance of therapeutic alliance in MBCT. Consequently, as therapeutic alliance may also serve to reduce risk of dropout (Swift & Greenberg, 2012), better therapeutic alliance might contribute to better adherence. This is of clinical importance, particularly in internet‐based MBCT, where dropout was shown to be higher than in face‐to‐face group MBCT (Compen et al., 2018).

### 4.1 Clinical implications

Completing MBCT is valuable for cancer patients as it results in long‐term reductions of psychological distress, and long‐term increases in positive mental health and quality of life (Cillessen et al., 2018). The findings that agreement on therapeutic goals is associated with better outcomes should be taken into account in the training of MBCT therapists in order to enhance the potential effect of MBCT. More attention could be paid, for example, on explaining the rationale of MBCT and engaging patients in the process or at least be willing to explore this.

## ETHICAL APPROVAL

All procedures performed in studies involving human participants were in accordance with the ethical standards of the institutional and/or national research committee and with the 1964 Helsinki declaration and its later amendments or comparable ethical standards.

## CONFLICTS OF INTEREST

The authors declare that there are no conflicts of interest.

## INFORMED CONSENT

Informed consent was obtained from all individual participants included in the study.
